# Roles of peroxisome proliferator‐activated receptors in hepatocellular carcinoma

**DOI:** 10.1111/jcmm.18042

**Published:** 2023-11-20

**Authors:** Yaqin Zhao, Huabing Tan, Xiaoyu Zhang, Jing Zhu

**Affiliations:** ^1^ Department of Abdominal Oncology, Cancer Center, West China Hospital Sichuan University Chengdu China; ^2^ Department of Infectious Diseases, Liver Disease Laboratory, Renmin Hospital Hubei University of Medicine Shiyan Hubei China; ^3^ Division of Gastrointestinal Surgery, Department of General Surgery The Affiliated Huai'an Hospital of Xuzhou Medical University Huai'an China; ^4^ Nanjing Drum Tower Hospital Nanjing China

**Keywords:** HCC, NAFLD, peroxisome proliferator‐activated receptor (PPAR), prognosis

## Abstract

Hepatocellular carcinoma (HCC), the main pathological type of liver cancer, is linked to risk factors such as viral hepatitis, alcohol intake and non‐alcoholic fatty liver disease (NAFLD). Recent advances have greatly improved our understanding that NAFLD is playing a major risk factor for HCC. Peroxisome proliferator‐activated receptors (PPARs) are a class of transcription factors divided into three subtypes: PPARα (PPARA), PPARδ/β (PPARD) and PPARγ (PPARG). As important nuclear receptors, PPARs are involved in many physiological processes, and PPARs can improve NAFLD by regulating lipid metabolism, accelerating fatty acid oxidation and inhibiting inflammation. In recent years, some studies have shown that PPARs can participate in the occurrence and development of HCC by regulating metabolic pathways. In addition, PPAR modulators have been reported to inhibit the proliferation and metastasis of HCC cells and can enhance the curative effect of conventional treatments. This article reviews the role of PPARs in the occurrence and development of HCC, as well as its value in the diagnosis, treatment and prognosis of HCC, in order to provide directions for future research.

## 1 INTRODUCTION

**FIGURE 1:**
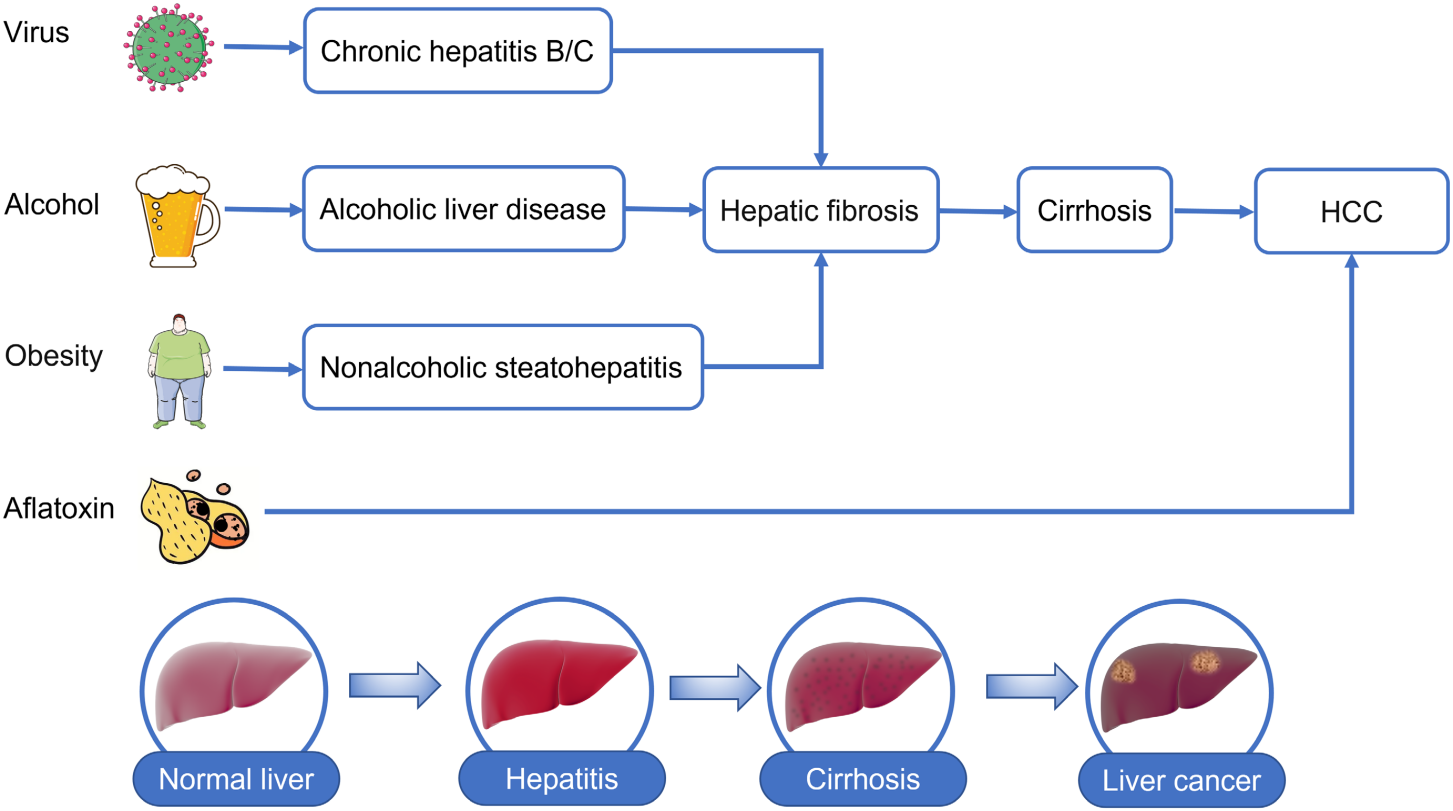
Main risk factors and development process of hepatocellular carcinoma.

Liver cancer is the sixth most common neoplasm and the third leading cause of cancer‐related death, with 906,000 incident cases and 830,000 deaths worldwide in 2020.^1^, ^2^, ^3^, ^4^ Hepatocellular carcinoma (HCC) represents about 75% ~ 85% of primary liver cancer. The risk factors of HCC include hepatitis virus infection, alcohol intake, aflatoxin exposure,^1^ etc. (Figure 1). In recent years, NAFLD, which is caused by obesity, diabetes and metabolic syndrome, has also attracted much attention as a risk factor for HCC. Despite advances in diagnosing and treating HCC, the overall 5‐year survival is still at 18%, and the recurrence rate of postoperative patients is up to 80%.^2^ Targeted therapy and immunotherapy bring new strategies to patients with advanced HCC. In patients with unresectable HCC, the results of the IMR‐150 trial suggested that the combination of atezolizumab with bevacizumab was superior to sorafenib in terms of overall survival (OS) and progression‐free survival, and the objective response rate increased to 27.3%.^5^ However, a significant number of patients may not be able to benefit from this.

**FIGURE 2:**
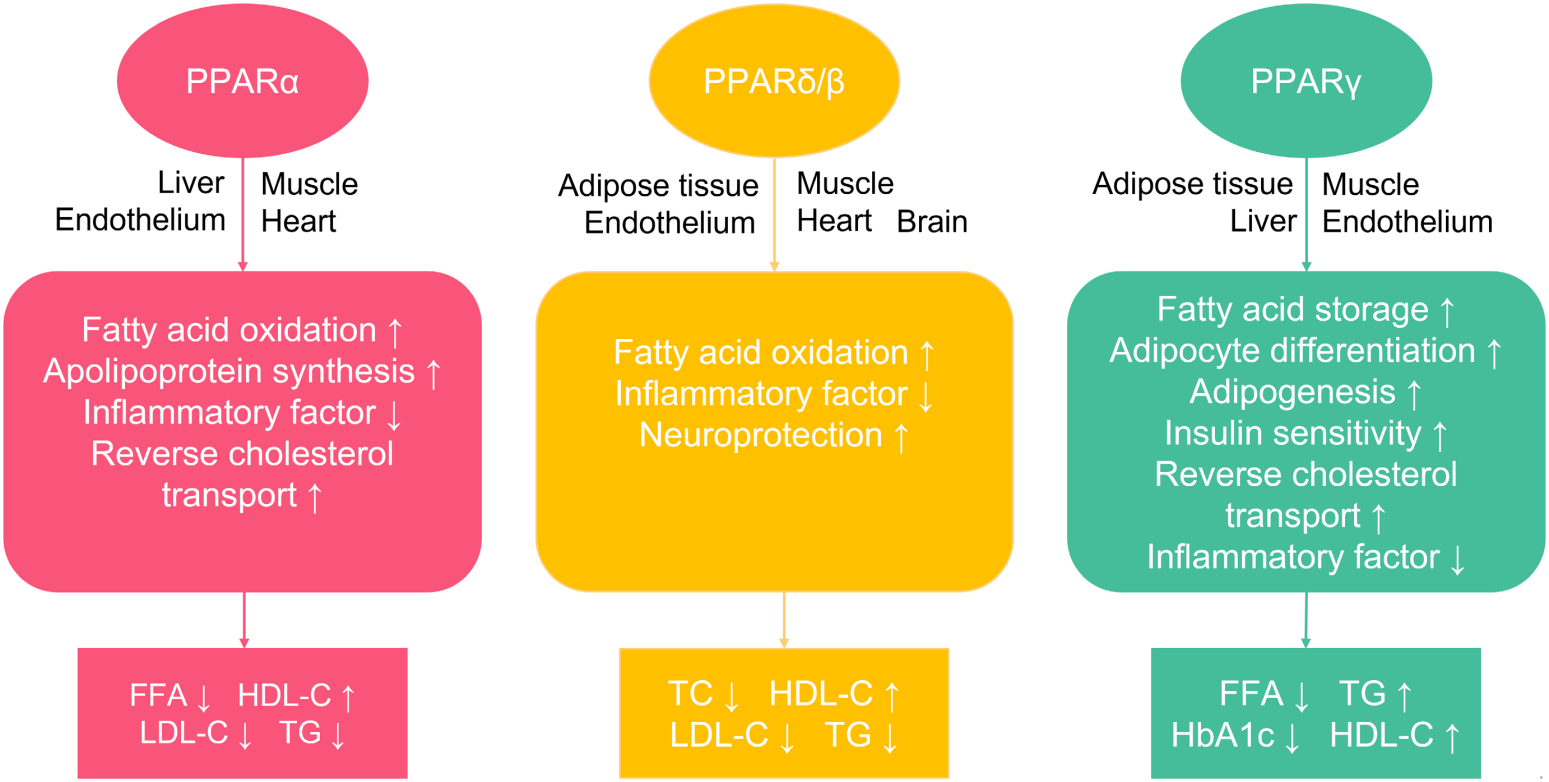
Distribution and function of three types of PPARs. FFA, free fatty acids; HDL‐C, high‐density lipoprotein cholesterol; LDL‐C, low‐density lipoprotein cholesterol; TC, total cholesterol; TG: triglyceride.

Peroxisome proliferator‐activated receptors (PPARs) are nuclear hormone receptors (NRs) that play a role in the regulation of immunological tolerance, metabolism and inflammation. The PPAR family consists of three isotypes: PPARα (PPARA), PPARδ/β (PPARD) and PPARγ (PPARG). These NRs are considered to be the master metabolic regulators which regulate many aspects of the body's energy homeostasis and cell fate^6^, ^7^ (Figure 2). PPARs have been observed in several tumour cells.^8^ Past research has shown that tumour formation and progression are often associated with PPAR signalling pathway abnormalities.^9^, ^10^ PPARα, expressed in the liver, heart, skeletal muscle and kidney, regulates fatty acid metabolism and lipid transport.^11^, ^12^ The ubiquitous expression of PPARβ/δ is related to fatty oxidation, energy consumption and lipid accumulation.^13^, ^14^, ^15^ PPARγ is expressed in adipose, muscle and macrophages and regulates adipogenesis and lipid storage.^8^, ^16^ According to existing studies, the three subtypes all have a double‐edged sword effect on tumour development.

The pathogenesis of HCC is complex. With increasing interest in cancer cell metabolism, this article aims to highlight the role of PPARs in hepatocarcinogenesis and the clinical significance of PPARs in the diagnosis, treatment and prognosis of HCC.

## 2 PPARs IN HEPATOCARCINOGENESIS

Cumulative evidence indicates that metabolic disorder is a potential mechanism of HCV‐related, alcohol‐related or non‐alcoholic fatty liver disease (NAFLD)‐related HCC.^17^, ^18^, ^19^, ^20^ Abnormal metabolism of fatty acid oxidation (FAO) in fatty liver disease may be one of the risk factors of HCC.^21^, ^22^ FAO metabolism mediated by PPARs has been verified by much evidence.^9^, ^23^ The decrease in PPAR signalling in the early stage of HCC leads to the loss of liver cell characteristics.^24^ The role of these three PPARs in HCC development will be elaborated below.

### 2.1 PPARα in hepatocarcinogenesis

PPARα, the first identified member of the PPAR family, is mainly expressed in tissues with high levels of fatty acid catabolism, such as the liver, skeletal muscle and heart. PPARα is closely associated with energy homeostasis and lipid metabolism.^25^, ^26^ Abnormal expression of PPARα may be related to a variety of metabolic disorders including fatty liver, alcoholic cardiomyopathy, atherosclerosis, etc. In tumours, PPARα is reported to be involved in mediating tumour proliferation, invasion and metastasis. The increased expression of PPARα can induce the transcription of long‐chain fatty acid‐CoA ligase 1 (ACSL1), thus promoting the synthesis of fatty acids.^27^ Chronic PPARα activation results in hepatocyte proliferation, hepatomegaly and ultimately HCC.^28^, ^29^ Kurokawa et al.^30^ reported that the expressions of PPARα mRNA and PPARα target genes mRNA increased in HCC tissues. Continuous activation of PPARα induces liver steatosis by increasing liver triglyceride synthesis, thus accelerating the development of HCC in patients with HCV infection.^31^, ^32^

PPARα was also reported to be regulated by oncogenes. Highly up‐regulated in liver cancer (HULC), astrocyte elevated gene‐1 (AEG‐1) and transcription factor EB (TFEB) promote lipogenesis via inhibiting PPARα in HCC.^26^, ^33^, ^34^, ^35^ 4‐PBA initiates cancer stem cells by activating the PPARα‐dependent Wnt5b‐Fzd5‐β‐catenin signalling pathway, promoting the early occurrence of HCC. In addition, 4‐PBA upregulates the expression of PPARα and directly binds to PPARα, enhancing its stability.^36^ MiR‐483‐5p can suppress the proliferation of HCC via targeting TIMP2 and PPARα, and downregulation of miR‐483‐5p is observed in human HCC tissues.^37^ MYC can enhance PPARα target gene Krt23 to promote the proliferation of hepatic cells and the occurrence of HCC.^38^ As a protein phosphatase, fructose‐1,6‐bisphosphatase 1 has the function of dephosphorylating histone H3T11, inhibiting the expression of PPARα‐mediated β‐oxidation gene and promoting the apoptosis induced by energy stress.^39^

Several pieces of evidence suggest the impaired activity of PPARα might contribute to HCC in patients with chronic HCV infection.^40^, ^41^ Mitochondrial fission inhibits FAO in HCC cells and promotes HCC growth and metastasis via down‐regulating the expression of PPARα and its target genes ACOX1 and CPT1A.^42^ LINC00467 negatively regulates the expression of miR‐9‐5p, alleviates the inhibitory effect of miR‐9‐5p on PPARα and suppresses the progression of HCC.^43^ CircRNA hsa_circ_0110102 can be used as a miR‐580‐5p sponge. MiR‐580‐5p upregulates the expression of C‐C chemokine ligand 2 (CCL2) by reducing the expression of PPARα in HCC cells. CCL2 in the tumour microenvironment activates the COX‐2/PGE2 pathway in macrophages in a p38 MAPK‐dependent manner via FoxO1, promoting the proliferation of HCC cells.^44^ Through activation of the p38 MAPK signalling pathway, CD147 downregulates PPARα and its transcription target genes CPT1A and ACOX1, thereby inhibiting fatty acids β‐oxidation. This process is involved in the proliferation and metastasis of HCC.^45^ Additionally, GO (Gene Ontology) and KEGG (Kyoto Encyclopedia of Genes and Genomes) indicated that the PPAR signalling pathway was downregulated in HCC. The knockout of ABCC6 can inhibit the activity of PPARα and prevent HCC cells from oxidative damage.^46^ PPARα also participates in the reprogramming of tumour metabolism. Adenosine triphosphate (ATGL) is reduced in HCC. Leo et al.^47^ discovered ATGL/PPARα/p300 axis was involved in the acetylation and stabilization of p53 in HCC cell lines, suggesting that ATGL might participate in the conversion of glycolytic oxidative metabolism in HCC cells.

### 2.2 PPARβ/δ in hepatocarcinogenesis

**FIGURE 3:**
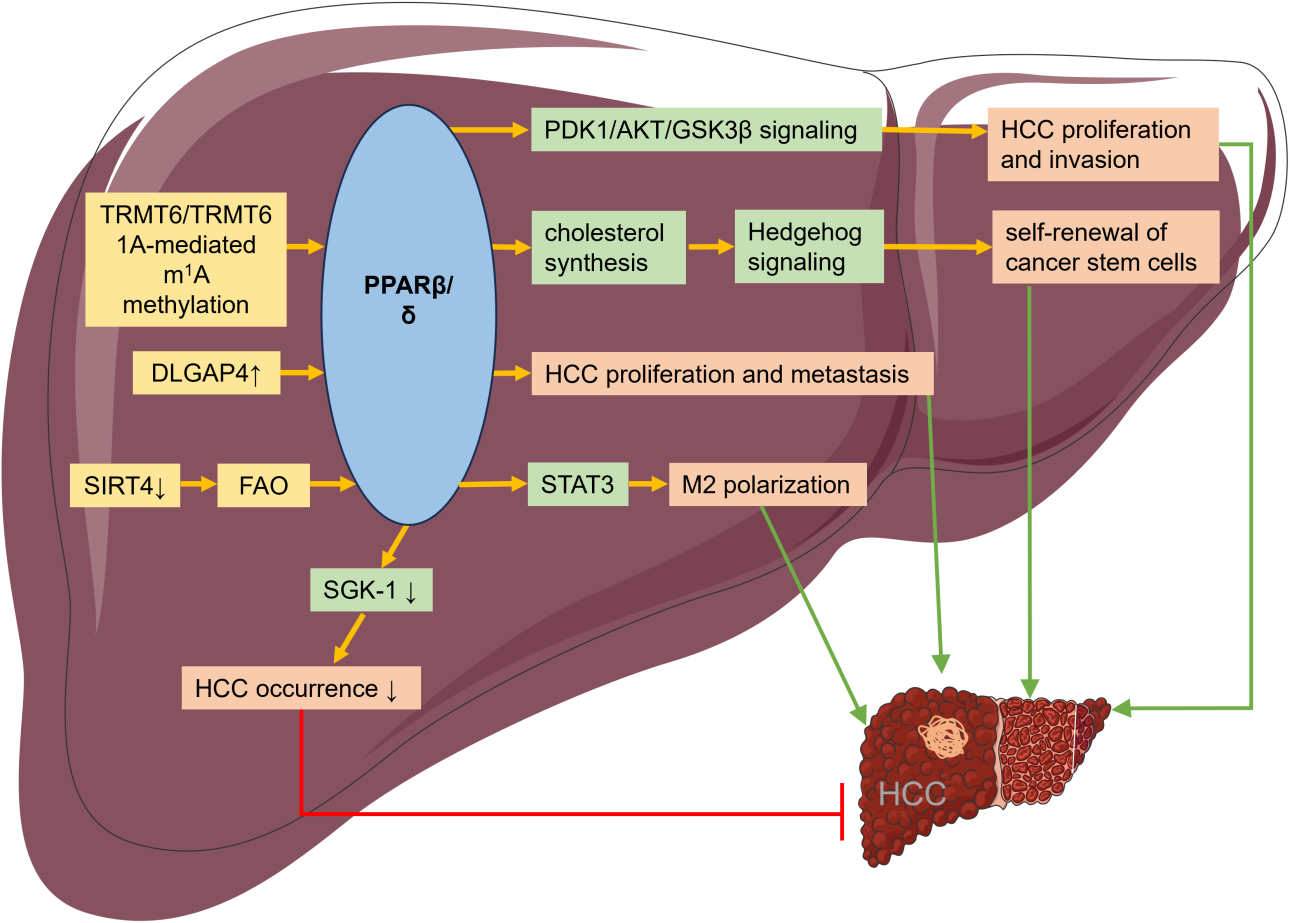
PPARβ/δ in hepatocarcinogenesis. AKT, protein kinase B; DLGAP4, disc large associated protein 4; FAO, fatty acid oxidation; GSK3β, glycogen synthase kinase 3 beta; PDK1, pyruvate dehydrogenase kinase 1; SGK‐1, serine/threonine‐protein kinase; SIRT4, Sirtuin 4; STAT3, signal transducer and activator of transcription 3; TRMT6, TRNA methyltransferase 6.

PPARβ/δ played a role in controlling liver carbohydrate and lipid metabolism. Because of regulating the expression of various FAO enzymes, PPARβ/δ is considered to be a key regulatory factor of tumour angiogenesis, invasion and migration.^48^, ^49^ In the occurrence and development of HCC, PPARβ/δ also plays an important role (Figure 3). Bioinformatics analysis showed that the expression of PPARβ/δ in HCC was significantly increased compared with normal liver tissue. Immunohistochemical results also confirmed this conclusion. Subsequent experiments indicated that PPARβ/δ, which was activated by a ligand, could induce HCC cells to proliferate and invade via PDK1/AKT/GSK3β signalling pathway.^50^

TRMT6/TRMT61A‐mediated m1A methylation promotes the translation of PPARβ/δ protein, triggering cholesterol synthesis to activate Hedgehog signalling, ultimately driving the self‐renewal of cancer stem cells and the occurrence of HCC.^51^ Disc large associated protein 4 (DLGAP4) is highly expressed in HCC cell lines and tissues. Overexpression of DLGAP4 increases the expression of PPARβ/δ and promotes the proliferation and metastasis of HCC.^52^ The expression of SIRT4 was reported significantly downregulated in HCC tissues. Further research found that the downregulation of SIRT4 in tumour‐related macrophages regulated macrophage replacement activation through the FAO‐PPARδ‐STAT3 axis and promoted HCC growth.^53^

In addition, PPARβ/δ showed tumour suppressor effect in some studies. For example, PPARβ/δ can directly bind to the oncogene serine/threonine‐protein kinase (SGK‐1), negatively regulate the expression of SGK‐1 and inhibit the HCC occurrence.^54^

### 2.3 PPARγ in hepatocarcinogenesis

PPARγ is enriched in the fatty acid metabolism pathway.^55^ PPARγ activation causes changes in glucose, fatty acid and glutamine metabolism, resulting in reactive oxygen species (ROS)‐mediated retinoblastoma protein hypophosphorylation and cell cycle arrest.^56^ PPARγ‐induced metabolic changes elicit direct effects on cell cycle progression, cellular differentiation and apoptosis.^56^, ^57^, ^58^ In the development of HCC, PPARγ plays a key role. PPARγ accelerates aerobic glycolysis and metabolic adaptations of lipogenesis in HCC via increasing lipid synthesis enzymes, including ATP citrate lyase, acetyl‐CoA carboxylase and fatty acid synthase.^59^, ^60^, ^61^, ^62^ It is also still controversial whether PPARγ plays an inhibiting or promoting role in the development of HCC.

The high expression of PPARγ can promote lipid synthesis and tumorigenesis in HCC.^63^, ^64^ FNDC5 induces M2 macrophage polarization through the PPARγ/NFκB/NLRP3 pathway, promoting the growth of HCC cells.^65^ XCT‐specific knockout in macrophages can inhibit M2‐type polarization, in which the SOCS3‐STAT6‐PPARγ pathway plays an important role.^66^ CMTM3 has been shown to promote HCC tumorigenesis, possibly related to the upregulation of PPARγ.^67^ LncRNA Ftx is elevated in HCC, which is related to bad pathologic features. LncRNA Ftx can regulate key glycolytic genes and promote HCC progression by activating the PPARγ pathway.^68^ MiR‐122 is highly expressed in the liver. Hepatitis B virus X protein can bind to PPARγ and inhibit the miR‐122 transcription.^69^

However, Yu et al.^70^ reported that the high expression of PPARγ significantly inhibited the activity of HCC cells and promoted the expression of caspase‐3, caspase‐7 and other caspases to increase cell apoptosis. PPARγ can directly target CITED2 and inhibit the progression of HCC.^71^ Han et al.^72^ found that hispidulin could up‐regulate the expression of PPARγ by activating the AMPK‐ERK signal pathway, thus inhibiting the proliferation and metastasis of HCC cells. Besides, activated PPARγ downregulates the expression of the pro‐metastatic genes matrix metalloproteinase 9 (MMP9) and MMP13 to suppress HCC metastasis.^73^ Cao et al.^74^ also proved that overexpression of PPARγ can inhibit the proliferation of HCC and induce apoptosis through downregulation of septin 2. The overexpression of PPARγ inhibits the invasion of HCC cells by upregulating the expression level of plasminogen activator inhibitor‐1 (PAI‐1).^75^ Winkler et al.^76^ revealed a network of 8 miRNAs and 54 target genes to combat liver fibrosis. The expression of this miRNA network was mainly controlled by PPARγ. MiR‐27a induces the proliferation of HCC cells by targeting the 3′‐untranslated region of PPARγ.^77^ The overexpression of PPARγ coactivator 1‐alpha (PPARγC1α) promotes HCC apoptosis, indicating that it might be a potential HCC therapeutic target.^78^

## 3 PPARs AND RELATED PATHWAYS AS DIAGNOSTIC BIOMARKERS

Researchers found that some differentially expressed long non‐coding RNAs, including RP11‐486O12.2, LINC01093, RP11‐273G15.2 and RP11‐863 K10.7, have been found to be rich in the PPAR signalling pathway. The combination of these 4 lncRNAs was used to distinguish HCC from adjacent normal tissues, and the area under the curve (AUC) was >0.85.^79^ The expression of HOTTIP was significantly up‐regulated in HCC. HOTTIP may play an important role in HCC by regulating PPAR signalling pathways. A meta‐analysis of 393 multi‐centre cases showed that the aggregated sensitivity and specificity were 0.88 and 0.59, respectively, and the AUC of summary receiver operating characteristic was 0.87.^80^

## 4 PPARs AND RELATED PATHWAYS AS PROGNOSTIC BIOMARKERS

PPAR members can be used as prognostic biomarkers of HCC (Table 1). Analysis of the expression of PPARα in 804 HCC specimens revealed that HCC patients with low cytoplasmic expression of PPARα had shorter OS and disease‐free survival (DFS). Multivariate Cox analysis showed that the expression of PPARα in the cytoplasm was a significant predictor of prognosis for OS.^81^ However, the study by Chen et al.^36^ reached the opposite conclusion. Their analysis of PPARα in 263 HCC specimens showed that the outcome was worse in those who were highly expressed PPARα, and PPARα was considered to be an independent predictor of recurrence and OS. Bioinformatics analysis showed a higher level of PPARγ expression in HCC compared to normal liver tissues, associated with poor prognosis. But an analysis of clinical samples came to the opposite conclusion, possibly related to the ethnicity of the samples.^55^

**TABLE 1:** Research progress of peroxisome proliferator‐activated receptors (PPAR) and its related pathways as prognostic biomarkers of hepatocellular carcinoma (HCC).

Biomarker	Test method	Outcome of prognosis	Ref.
Types of PPAR			
Cytoplasmic PPARα↓	IHC	Recurrence↑, OS↓, DFS↓	81
PPARα↑	IHC	Recurrence↑, OS↓	36
PPARγ↑	IHC	OS↑	55
USP22/PPARγ/ACACA↑	Expression profiling	OS↓	64
PPARγ↑	Expression profiling	OS↓	82
PPARγ↓	IHC	Macroscopic vascular invasion↑, DFS↓, OS↓	83
Altered PPAR activity	RNA sequencing	OS↓	84
PPAR signalling pathway and its related genes			
ABCC6↓	Expression profiling	OS↑ in early‐stage HCC	46
MMP‐1↑	IHC	OS↓, DFS↓	85
HMGCS2↓	IHC	OS↓, DFS↓	86
SLC27A↓	IHC	OS↓	87
ALDH7A1↓	Expression profiling	OS↓	88
KIAA1522↑	Expression profiling	OS↓	89
ACOX2↓	Proteomics examination	OS↓	90
Risk scores based on 5 miRNAs ↑	Expression profiling	OS↓	91

Abbreviations: ACOX2, acyl‐CoA oxidase 2; DFS, disease‐free survival; IHC, immunohistochemistry; MMP‐1, matrix metallopeptidase 1; HMGCS2, mitochondrial 3‐hydroxy‐3‐methylglutaryl‐CoA synthase; OS, overall survival; USP22, ubiquitin‐specific protease 22.

Another study found that in the HCC cohort, the simultaneous high expressions of USP22/PPARγ/ACACA were associated with poor OS.^64^ Through bioinformatics analysis and experimental validation, Wang et al.^82^ have discovered that there is a positive correlation between PPARγ and TP53 mutation in HCC, and the high expression of PPARγ indicates a poor prognosis. Immunohistochemistry was used to detect the expression of PPARγ in 83 HCC tissues, and the relationship between the PPARγ expression and clinical pathology was analysed. These findings indicated that low expression of PPARγ was an independent predictor of more macroscopic vascular invasion.^83^ RNA sequencing was used to evaluate the expression of PPAR in 52 patients undergoing hepatectomy for HCC. The results showed that altered PPAR activity was associated with reduced OS.^84^

The PPAR signalling pathway has been identified as a prognostic feature in HCC, and it has clinical significance in searching for PPAR target genes for prognosis.^92^, ^93^ PPAR signalling pathway was downregulated in HCC, associated with unfavourable OS and recurrence‐free survival (RFS).^46^, ^94^ MMP1, HMGCS2 and SLC27A5 participate in the PPAR signalling pathway. MMP1 is known to be a risk factor for cancer development,^95^ and its prognostic value in HCC has also been confirmed. The prognosis of HCC patients with high expression of MMP‐1 is poor. Multivariate analysis found that MMP‐1 was an independent prognostic factor for OS and DFS.^85^ HMGCS2 is confirmed as a tumour suppressor,^96^ and low expression of HMGCS2 is associated with poor OS and DFS of HCC patients.^86^ Xu et al.^87^ also indicated that MMP1 had a negative effect on HCC patients' outcomes, while HMGCS2 and SLC27A might be beneficial to prognosis. The combination of these three genes could effectively predict the prognosis of HCC.

The deletion of ALDH7A1 leads to a decrease in endogenous PPARα ligands and a reduction in PPAR transcriptional activity. Low expression of ALDH7A1 protein is related to poor prognosis in HCC patients.^88^ KIAA1522 expression in HCC tumour tissue is elevated and indicates poor prognosis, possibly due to inhibition of PPAR signalling pathways.^89^ Through data‐independent acquisition, quantitative proteomics, bioinformatics methods and experimental validation, Zhang et al.^90^ revealed that the low expression of acyl‐CoA oxidase 2 (ACOX2) in HCC indicated an unfavourable prognosis. Overexpression of ACOX2 could inhibit HCC proliferation and metastasis through the PPARα pathway. Liu et al.^91^ conducted training and validation based on 322 HCC patients in the Cancer Genome Atlas database. Risk scores were developed for the 5 miRNAs identified in the training set (has‐miR‐301a, has‐miR‐132, has‐miR‐212, has‐miR‐489 and has‐miR‐1468). Scores were computed for every patient in the testing set. Patients with high risk had a poorer prognosis than those with low risk. Subsequent research indicated that the target genes of these 5 miRNAs were highly enriched in the PPAR signalling pathway.

## 5 PPAR AND RELATED PATHWAYS AS PROMISING THERAPEUTIC TARGETS

Metabolic‐associated fatty liver disease is characterized by liver steatosis, inflammation and hepatocyte ballooning, leading to advanced fibrosis, cirrhosis and eventually HCC.^97^, ^98^ The three isoforms, PPARα, PPARß/δ and PPARγ, are expressed in various compartments of the liver, making them an attractive target for treatment.^99^, ^100^ Several studies have been conducted on various drugs targeting single, two or three PPAR subtypes in HCC (Table 2).

**TABLE 2:** The potential role of peroxisome proliferator‐activated receptors (PPAR) modulators in the treatment of hepatocellular carcinoma (HCC).

Type of PPAR Modulator	Effect on HCC	Ref.
PPARα agonist		
Fenofibrate	↓AKT phosphorylation, ↓HCC	101
Pemafibrate	Combined with tofogliflozin, ↓IRE1α‐XBP1‐PHLD3A pathway, ↓ER stress, ↓HCC	102
Gallic acid	AMPK‐ACC‐PPARα pathway, ↓HCC	103
PPARβ agonist		
GW501516	↑cPLA2α/COX‐2/PGE2 pathway, ↑EGFR and Akt phosphorylation, ↑HCC	104
	↑Cpt1 and Tgfβ1 mRNA, ↓cyclins D1 and Cyclin E1, ↓HCC	105
GW0742	↓steatosis, ↓proliferation, ↑apoptosis, ↓HCC	106
PPARγ agonist		
Telmisartan	↑NF‐κB‐TAK1‐ERK1/2 axis, ↓HCC	107
Celecoxib	↑PTEN, ↓Akt, ↓HCC stem cells	108
Pioglitazone	Combined with capecitabine and rofecoxib in a phase II clinical study, the disease control rate: 79%	109
Simvastatin	↓HIF‐1α/PPAR‐γ/PKM2‐mediated glycolysis, re‐sensitizes HCC cells to sorafenib	110
Rosiglitazone	↑CDH1, SYK, TIMP3 and RB1, ↓MMP9, MMP13, HGF and heparinase, ↓HCC	73
PPARγ antagonist		
T0070907, GW9662	↑vimentin cleavage, ↓high‐grade HCC	111
Dual PPAR modulators		
Dual PPARɑ/γ agonist		
Oroxyloside	↓glucose catabolism, ↑fatty acids oxidation, ↑ROS, ↓HCC cell cycle	112
Saroglitazar	↓steatosis, fibrosis and inflammation, ↓NASH → HCC	113

Abbreviations: Cpt1, carnitine palmitoyltransferase 1; ERK, extracellular signal‐regulated kinase; HGF, hepatocyte growth factor; HIF‐1α, hypoxia‐inducible factor‐1α; MMP, matrix metallopeptidase; NFκB, nuclear transcription factor kappa‐B; PKM2, pyruvate kinase type M2; RB1, retinoblastoma 1; ROS, reactive oxygen species; SYK, spleen tyrosine kinase; TAK1, transforming growth factor beta‐activated kinase 1; TIMP3, TIMP metallopeptidase inhibitor 3.

### 5.1 PPARα modulators

The PPARα agonist, fenofibrate, can cause inhibitory effects on HCC.^101^ Combined therapy with PPARα modulators pemafibrate and sodium‐glucose co‐transport 2 inhibitor tofogliflozin inhibits IRE1α‐XBP1‐PHLD3A pathway, reduces endoplasmic reticulum stress‐induced liver damage and has potential to treat NASH‐related HCC.^102^ Zhang and his colleagues determined that the physiochemical gallic acid (GA) played a key role in the progression of NAFLD to HCC. The interaction between GA and AMPKα inactivated the ACC‐PPARα axis. The AMPK‐ACC‐PPARα axis is a possible target for NAFLD treatment.^103^

### 5.2 PPARβ modulators

PPARβ modulators have shown therapeutic potential in a variety of tumour and non‐tumour diseases. In nasopharyngeal carcinoma, the PPARβ/δ agonist GW501516 suppresses the development of undifferentiated nasopharyngeal carcinoma by regulating miR‐206.^114^ Zuo et al.^115^ found that upregulation of PPAR‐δ/β significantly enhanced the susceptibility to colon tumorigenesis, suggesting that great caution is needed in developing therapeutics to activate PPAR‐δ/β for noncancerous diseases.

PPARδ agonist GW501516 can increase the expression of PPARβ. PPARδ interacts with the cPLA2α/COX‐2/PGE2 signalling pathway, enhances the phosphorylation of EGFR and Akt and promotes the proliferation of HCC cells.^104^ In contrast, previous studies by Michele et al. demonstrated that GW501516 inhibited Hepa 1‐6 cell proliferation.^105^ Lu et al.^116^ designed a sequential drug delivery strategy of carvedilol‐loaded star‐like nanozyme (Au NS@CAR‐HA). Au NS@CAR‐HA could inhibit the activity of PPARβ and c‐Jun N‐terminal kinase and reduce the proliferation and activation of hepatic stellate cells, thereby attenuating liver fibrosis. PPARβ/δ agonist GW0742 promotes the activation of PPARβ/δ ligand to reduce the occurrence of HCC in HBV transgenic mice via inhibiting steatosis and proliferation and inducing apoptosis.^106^

### 5.3 PPARγ modulators

PPARγ agonist has the potential to treat hepatic disorders such as inflammation, fibrosis, NAFLD and HCC.^16^ PPARγ agonist is associated with improved prognosis of HCC.^117^ Metabolism alterations caused by PPARγ result in significantly elevated ROS, leading to a rapid decrease in the phosphorylation of retinoblastoma protein and cell cycle arrest.^56^ In a mouse model of N‐nitrosodiethylamine‐induced HCC, telmisartan can activate PPARγ and regulate the NF‐κB‐TAK1‐ERK1/2 axis, inhibiting tumour proliferation and metastasis.^107^ Furthermore, PPARγ ligand pioglitazone is recommended for selected NAFLD patients by the European‐ and American Association Guideline for the Study of the Liver.^118^, ^119^ Celecoxib can activate PPARγ and up‐regulate PTEN, thereby inhibiting Akt and disrupting the expansion of HCC stem cells.^108^ The safety and effectiveness of combination therapy with capecitabine, pioglitazone and rofecoxib for advanced HCC are assessed in a phase II clinical study, and the disease control rate is 79%.^109^ The inhibitory effects of sorafenib on HCC may be associated with the PPAR signalling pathway.^120^ Simvastatin has been shown to suppress hypoxia‐inducible factor‐1α/PPARγ/pyruvate kinase type M2‐mediated glycolysis to re‐sensitize HCC cells to sorafenib.^110^ PPARγ agonist rosiglitazone inhibits HCC migration by directly binding PPAR to the heparanase promoter, reducing heparanase gene transcription in HCC.^73^ PPARγ antagonists can inhibit the invasion of high‐grade HCC cells by inducing vimentin cleavage.^111^ In a word, PPARγ is a potential therapeutic target for HCC.^121^

### 5.4 Dual PPAR modulators

In preclinical models of NAFLD, the dual PPARα/β agonist GFT505 improves steatosis, inflammation and fibrosis.^122^ The carboxylesterase 1 (CES1)‐PPARα/γ‐SCD axis may participate in the resistance of HCC cells to cisplatin by interfering with lipid signalling pathways. Cisplatin combined with CES1 inhibitors can effectively slow down the growth of HCC xenografted mice.^123^ Additionally, a dual PPARɑ/γ agonist oroxyloside can inhibit the HCC cell proliferation through metabolic transformation.^112^ Giri et al.^113^ reported that saroglitazar, a PPARα/γ dual agonist, could effectively prevent NASH from developing into liver cancer by inhibiting steatosis, fibrosis and inflammation in rodent models. Saroglitazar is authorized to treat NASH in India. chiglitazar sodium, a PPAR agonist, activates PPARα, γ, δ, reduces insulin resistance, effectively controls blood sugar and improves the disorder of blood lipid and energy metabolism often associated with T2DM patients, with good safety. However, whether chiglitazar sodium can be used in the treatment of HCC needs further study.

PPAR modulators have shown therapeutic effects on HCC in multiple preclinical and clinical studies. However, there is significant uncertainty regarding the side effects of PPAR modulators. Past research has suggested a positive association between the long‐term use of rosiglitazone in type 2 diabetic patients and the incidence of bladder cancer,^124^ but the latest study showed that there is no correlation between the two^125^ and that pioglitazone is associated with the increased risk of bladder cancer.^126^ Furthermore, the major adverse reactions of PPAR modulators include an increased risk of adverse cardiovascular events and possible hepatorenal toxicity.^127^

## 6 RECENT CHALLENGES AND FUTURE PERSPECTIVES

Non‐alcoholic fatty liver disease, which accounts for about 25% worldwide, is one of the major risk factors for hepatic cirrhosis and HCC.^128^, ^129^ An epidemiological study showed that among 5098 HCC patients, 35.6% were mainly attributable to NAFLD. Compared with patients with hepatitis C‐related HCC, patients with NAFLD‐related HCC had a lower early diagnosis rate and poorer OS.^130^ Halting the progression of NAFLD may be a challenging problem in the prevention of NAFLD‐associated HCC.

Metabolic disorder is a key factor in the occurrence and development of HCC.^97^, ^131^ Whether the metabolic disorder is the cause or result of HCC is still uncertain. The loss of hepatic cell‐specific glucose isomerase fructose 1, 6‐diphosphatase 1 destroys the liver metabolic homeostasis, leading to the ageing of hepatic stellate cells and steatosis, thus exacerbating tumour progression.^132^ In addition, the development of HCC causes metabolic changes. The rapid growth of cancer cells requires more metabolic supply. Some drugs targeting the metabolic pathways of HCC have shown anti‐tumour effects in preclinical studies.^97^ Targeting mIDH1 or SLC7A5 may be a promising therapeutic option for HCC by interfering with metabolism, but it still needs further evidence exactly its safety and efficacy.

PPARs are important for many cellular functions, such as lipid metabolism, glucose balance, inflammatory response and cellular proliferation. It has been known that PPARs participate in the pathophysiological process of NAFLD and HCC, but whether PPARs play the role of tumour suppressor or tumour promoter in HCC is not very clear, and the three subtypes of PPARs seem to have dual roles.

PPAR modulators have shown good effects in preclinical and clinical studies by inducing HCC cell apoptosis and inhibiting HCC cell invasion and metastasis. Most HCC patients are resistant to immune checkpoint blockades (ICB). Xiong et al.^133^ found that 40% of HCC patients resistant to pembrolizumab had high expression of PPARγ in the tumour. Mechanistically, up‐regulated PPARγ activated vascular endothelial growth factor‐A to induce myeloid‐derived suppressor cell expansion suppression of CD8+ T cell function. The use of a selective PPARγ antagonist triggered a switch from an immunosuppressive to an immunostimulatory TME and resensitized tumours to ICB therapy. Combining ICB therapy with PPARγ antagonists may provide a new strategy against immunotherapy resistance in HCC. In addition to PPARγ antagonists, targeting PPARβ or PPARα may also be helpful to improve the immunotherapy effect. PPARδ has been found to play an important role in the development and immunosuppressive activity of IL‐10+ regulatory B cells. Inhibition of PPARδ in B cells can significantly improve the therapeutic effect of anti‐PD‐1 antibody on melanoma.^134^ A phase Ib/II clinical result showed that in patients with unresectable or metastatic HCC, compared with the standard treatment scheme of atezolizumab combined with bevacizumab, the addition of small molecular PPARa antagonist TPST‐1120 showed a clinically significant improvement, with the unconfirmed ORR improving by 74.4% (30% vs 17.2%) and the confirmed ORR improving by 69.9% (17.5%).^135^ More clinical trials are expected to evaluate the effectiveness and safety of PPAR modulator combined with immunotherapy. The United States Food and Drug Administration has approved sorafenib and lenvatinib as first‐line treatment options for HCC. A recent study revealed that sorafenib targets SCD1 through the ATP/AMPK/mTOR/SREBP1 pathway, thereby destroying lipogenesis and triggering HCC cell death,^136^ and a reduced mitochondrial biogenesis is observed in sorafenib‐resistant cells, mechanically by the accelerated degradation of PPARγ coactivator 1β.^137^ Four genes in the PPAR signalling pathway (RXRB, NR1H3, CYP8B1 and SCD) have been identified as sorafenib resistance‐related genes, and SCD has been experimentally verified. Combining SCD inhibitors with sorafenib is likely to be a promising therapeutic option.^138^ Regulating signal pathways related to cell metabolism may enhance the efficacy of tyrosine kinase inhibitors in HCC cells. Targeting PPARs may also serve as an additional option for the treatment of HCC when combined with sorafenib or lenvatinib. Whether the multi‐target PPAR modulator has fewer side effects and better curative effect than the single‐target PPAR modulator in the treatment of HCC needs further exploration. It is necessary to deeply study the role of the PPAR signalling pathway in HCC, explore new targets and find or develop low‐toxicity and high‐efficiency drugs.

Additionally, intestinal microbiota may be involved in the occurrence of HCC.^137^ Whether PPAR can directly affect the pathogenesis and progression of HCC by regulating intestinal microbiota requires further research.^139^ Although many researchers have paid attention to the regulation function of PPAR in HCC, little has been done to investigate the effect of PPAR on immune cells and stromal cells in the tumour immune microenvironment, which needs further study.

## 7 CONCLUSION

This article reviews the promotion or inhibition of the three subtypes of PPAR in the development of HCC and expounds their great potential value as diagnostic or prognostic markers. In addition, PPAR may be a key therapeutic target. Rational use of PPAR modulators combined with existing treatment options may improve the efficacy of HCC treatment and bring new hope to HCC patients.

## AUTHOR CONTRIBUTIONS

**Yaqin Zhao:** Writing – review and editing (equal). **Huabing Tan:** Writing – review and editing (equal). **Xiaoyu Zhang:** Writing – review and editing (equal). **Jing Zhu:** Writing – review and editing (supporting).

## CONFLICT OF INTEREST STATEMENT

The authors declare that they have no conflicts of interest.

## Data Availability

Data sharing is not applicable to this article as no new data were created or analyzed in this study.
